# Circulating levels of soluble MER in lupus reflect M2c activation of monocytes/macrophages, autoantibody specificities and disease activity

**DOI:** 10.1186/ar4407

**Published:** 2013-12-10

**Authors:** Gaetano Zizzo, Justus Guerrieri, Lindsay M Dittman, Joan T Merrill, Philip L Cohen

**Affiliations:** 1 Section of Rheumatology, Department of Medicine, Temple University School of Medicine, 3322 N Broad Street, Philadelphia, PA 19140, USA; 2 Temple Autoimmunity Center, Temple University School of Medicine, 3500 N Broad Street, Philadelphia, PA 19140, USA; 3 Oklahoma Medical Research Foundation, 825 NE 13th Street, Oklahoma City, OK 73104, USA

## Abstract

**Introduction:**

Systemic lupus erythematosus (SLE) is characterized by impaired efferocytosis and aberrant activation of innate immunity. We asked if shedding of MER receptor tyrosine kinase (MerTK) and AXL into soluble (s) ectodomains was related to immunological and clinical aspects of SLE.

**Methods:**

Levels of sMER and sAXL in the plasma of 107 SLE patients and 45 matched controls were measured by ELISA. In 40 consecutive SLE patients, we examined potential correlations between either sMER or sAXL and plasma levels of sCD163, a marker of M2 activation. All three soluble receptors were measured in supernatants of monocytes/macrophages cultured in various immunological conditions. Membrane expression of MerTK, AXL and CD163 was assessed by flow cytometry.

**Results:**

Both sMER and sAXL were associated with anti-chromatin and anti-phospholipid autoantibodies, and with hematological and renal involvement. However, sMER and sAXL did not significantly correlate with each other; sAXL correlated with growth arrest-specific 6 (Gas6), whereas sMER correlated with reduced free protein S (PROS) levels. Only sMER showed significant associations with lupus-specific anti-dsDNA, anti-Sm, anti-ribonucleoprotein (anti-RNP) and anti-Ro60 autoantibodies. Strong correlations with disease activity indices (Systemic Lupus Erythematosus Disease Activity Index (SLEDAI), complement reduction, titer of circulating anti-dsDNA) were found for sMER, not for sAXL. Patients with active SLEDAI, nephritis, anti-dsDNA and anti-Ro60 positivity showed higher levels of sMER compared to controls. Levels of sMER, not sAXL, correlated with sCD163 levels, and these correlated with SLEDAI. Production of sMER and sCD163 occurred under “M2c” polarizing conditions, whereas sAXL was released upon type-I IFN exposure.

**Conclusions:**

Alterations in homeostasis of anti-inflammatory and efferocytic “M2c” monocytes/macrophages may have a role in immunopathogenesis of SLE.

## Introduction

Systemic lupus erythematosus (SLE) is an autoimmune disease characterized by defective phagocytosis of apoptotic cells (ACs) [1]. Accumulation and presentation of AC-derived nuclear and membrane autoantigens in lymphoid organs are believed to drive the activation of autoreactive B and T cells, leading to production of antinuclear and antiphospholipoprotein autoantibodies. Immune complexes containing nuclear antigens and antibody-opsonized ACs bind to Toll-like receptors (TLRs) and immunoglobulin G Fc receptors (FcγRs) on innate immune cells, provoking aberrant production of type I interferons α and β (IFN-α/β) and proinflammatory cytokines [2,3]. Additionally, noningested ACs undergo secondary necrosis, which fuels ongoing innate inflammation by amplifying TLR activation and oxidative burst [4,5].

Clearance of ACs is crucial for resolution of inflammation and maintenance of immune tolerance [6]. In healthy individuals, discrete populations of phagocytes, called M2c (CD163^+^) macrophages, are designated to promptly remove ACs, including activated immune cells undergoing apoptosis [7-9]. Moreover, the physiologic engulfment of ACs is associated with macrophage release of anti-inflammatory cytokines [6].

The Mer receptor tyrosine kinase (MerTK), which belongs to the family of Tyro3, Axl and MerTK (TAM) receptors (TAMRs), is required for the efficient clearance of ACs exerted by M2c monocytes/macrophages [9], participates in immune regulation by stimulating interleukin 10 (IL-10) secretion [9-11] and is involved in restoration of tissue homeostasis after inflammatory processes as well as in the maintenance of central and peripheral tolerance [11-14]. Another member of the TAMR family, Axl, is importantly involved in the deactivation of innate immune cells stimulated by TLR agonists and type I IFNs through recruitment of suppressors of cytokine signaling 1 and 3 and the transcriptional repressor Twist [15-17]. Both MerTK and Axl inhibit TLR-induced activation of nuclear factor κB (NF-κB) transcription factors and production of proinflammatory cytokines such as tumor necrosis factor α (TNF-α) and IL-6 [9-11,15-17].

Both the Mer and Axl receptors are susceptible to posttranslational regulation through ectodomain shedding mediated by a disintegrin and metalloprotease domain (ADAM) metallopeptidases [18-20]. In the present study, we measured the soluble (s) ectodomains sMer and sAxl in the circulation of SLE patients and matched healthy individuals with the aim of investigating how these molecules relate to clinical, laboratory and immunological profiles of SLE; how they are related to each other and to the TAMR ligands growth arrest–specific 6 (Gas6) and reduced free Protein S (ProS); and under what immunological conditions they are produced. We found that plasma levels of both sMer and sAxl were related to general aspects of systemic autoimmunity and were associated with hematological and renal involvement. However, sMer and sAxl did not significantly correlate with each other. Compared to sAxl, sMer showed closer relations with specific aspects of SLE immunopathogenesis, such as production of lupus-specific autoantibodies and reduction of free ProS in circulation. Strong correlations with disease activity indices were found for sMer, but not for sAxl. Patients with signs of active SLE showed higher levels of sMer compared to matched controls. Remarkably, sMer levels in SLE patients directly correlated with circulating levels of sCD163, a well-known marker of M2 activation, and sCD163 levels correlated with Systemic Lupus Erythematosus Disease Activity Index (SLEDAI) score. In fact, sMer and sCD163 were found to be released under the same M2c polarizing conditions. Production of sAxl was instead enhanced in the presence of IFN-α or IFN-β, and plasma concentrations of sAxl in SLE patients correlated with increased Gas6 levels. Our data highlight, through the study of sMer and sCD163, a strict relationship between SLE pathogenesis and homeostasis of anti-inflammatory and efferocytic M2c monocytes/macrophages. We also provide indirect proof, through the study of sAxl, that type I IFN stimulation plays a role in the development of systemic autoimmunity but does not seem to be closely related to SLE disease activity. Whether augmented ectodomain shedding of membrane receptors reflects increased turnover and/or activation of the respective pathways or rather contributes to their dysfunction and/or inhibition remains to be clarified.

## Methods

### Participants

Plasma samples from 107 SLE patients participating in the Oklahoma Cohort for Rheumatic Disease were studied. All patients satisfied at least four of the 1982 revised American Rheumatism Association criteria for SLE [21]. Forty-five of these patients were matched to healthy controls by age, gender and ethnicity. Clinical and laboratory data were registered into a database which included no personal identifiers. The characteristics of the patients and the controls enrolled are reported in Table 1. Heparinized plasma samples were collected and stored at −70°C immediately after collection. Disease activity was scored using the SLEDAI and the British Isles Lupus Assessment Group (BILAG) index [22,23]. Levels of complement fractions C3 and C4 were determined by immunoturbidity. Total 50% hemolytic complement (CH_50_) activity was calculated by using a liposome immunoassay. Antinuclear antibodies were detected by indirect immunofluorescence. The *Crithidia luciliae* test was used for detection of anti-double-stranded DNA (anti-dsDNA). Antiextractable nuclear antigen (anti-ENA) autoantibodies were measured by Ouchterlony double-immunodiffusion. Prior to participation, all participants gave their informed consent to donate their blood samples. The study was approved by the institutional review boards of the Oklahoma Medical Research Foundation and Temple University.

**Table 1:** **Demographic, clinical and immunological characteristics of the patients**^**a**^

**Characteristics**	**Total patients** **(*N*** **= 107)**	**Matched patients** **(*N*** **= 45)**	**Matched controls** **(*N*** **= 45)**
Age (years)	39.6 ± 14.1	46.7 ± 15.4	45.4 ± 15.9
Sex (F:M ratio)	3.9:1	3.1:1	3.1:1
Ethnicity			
Caucasian (%)	88.9	86.9	86.9
African (%)	6.7	6.5	6.5
Asian (%)	2.2	3.7	3.7
American Indian (%)	2.2	2.8	2.8
ACR total (number of criteria met)	5.51 ± 1.69	5.52 ± 1.69	
Antichromatin Ab	32.7	28.9	
Anti-dsDNA Ab (*Crithidia luciliae* test) (%)	27.1	33.3	
Anticardiolipin IgG Ab (%)	38.3	28.9	
LAC (%)	13.1	11.1	
Anti-Smith Ab (%)	18.7	11.1	
Anti-RNP Ab (%)	20.6	17.8	
Anti-Ro/SSA (Ro52/60 kDa) Ab (%)	38.3 (21.5/34.6)	31.1 (17.8/28.9)	
Anti-La/SSB (anti-Ro60 + anti-La) Ab (%)	16.8 (15.0)	15.6 (13.3)	
CH_50_ <40 (U/ml)	51.4	55.6	
C4 <16 (mg/dl)	35.5	44.4	
SLEDAI score	5.29 ± 4.20	5.02 ± 3.26	
Renal involvement (%)	13.1	24.4	
(BILAG grades A to C)			
Mucocutaneous involvement (%)	68.2	57.8	
Musculoskeletal involvement (%)	77.6	66.7	
Cardiovascular/respiratory involvement (%)	14	15.6	
Hematological involvement (%)	37.4	31.1	
Vasculitis (%)	53.3	53.3	
Neurological involvement (%)	3.7	4.4	
BILAG total score	6.17 ± 4.53	5.70 ± 3.93	
Gas6 plasma levels (ng/ml)	18.81 ± 8.67	17.64 ± 7.10	15.89 ± 6.88
Free protein S plasma levels (μg/ml)	6.78 ± 2.36	6.37 ± 1.79	6.91 ± 1.74

^a^Ab, antibody; ACR total, number of American Rheumatism Association 1982 revised criteria for classification of systemic lupus erythematosus; BILAG, British Isles Lupus Assessment Group index; C4, complement fraction 4; CH_50_, 50% hemolytic complement activity of serum; dsDNA, double-stranded DNA; Gas6, growth arrest–specific 6; IgG, immunoglobulin G; LAC, lupus anticoagulant; RNP, ribonucleoprotein; SLEDAI, Systemic Lupus Erythematosus Disease Activity Index; SSA, Sjögren’s syndrome antigen A; SSB, Sjögren’s syndrome antigen B.

### Cell cultures

Monocytes from buffy coats of healthy blood donors were isolated with Ficoll-Paque PLUS gradient (GE Healthcare Life Sciences, Pittsburgh, PA, USA) and by magnetic separation using a kit for human monocyte enrichment by negative selection (EasySep cell isolation platform; STEMCELL Technologies, Vancouver, BC, Canada) according to the manufacturer’s instructions. The purity of CD14^+^ cells was >90% as assessed by flow cytometry. CD14^+^ cells were cultured for 3 days at 0.8 × 10^6^ cells/ml in 24-well plates containing serum-free X-VIVO 15 medium (Lonza, Walkersville, MD, USA) in the presence or absence of macrophage colony-stimulating factor (M-CSF) (50 ng/ml; PeproTech, Rocky Hill, NJ, USA), granulocyte macrophage colony-stimulating factor (GM-CSF) (100 ng/ml; PeproTech), IL-10 (50 ng/ml; PeproTech), IFN-α (3,000 U/ml; Novus Biologicals, Littleton, CO, USA), IFN-β (3,000 U/ml; PeproTech), IFN-γ (2.5 ng/ml; R&D Systems, Minneapolis, MN, USA), IL-4 (20 ng/ml; Novus Biologicals), IL-17 (100 ng/ml; R&D Systems) or dexamethasone (100 nM; Sigma-Aldrich). When specified, on day 2, cells were coincubated with lipopolysaccharide (LPS) (100 ng/ml; Sigma-Aldrich) for the remaining 24 hours. Cells were then harvested by centrifugation. Supernatants were collected and immediately stored at −20°C for a few days before being tested by enzyme-linked immunosorbent assay (ELISA). Pellets were resuspended in phosphate-buffered saline (PBS) and immediately analyzed by flow cytometry.

### Enzyme-linked immunosorbent assay

Plasma concentrations of sAxl, sMer and sCD163 were measured by sandwich ELISA according to standard procedures [24]. Briefly, 96-well plates were precoated overnight with a capture antibody. Heparinized plasma samples were diluted 1:10 in PBS containing 1% bovine serum albumin (BSA) and applied to precoated plates in duplicate. Serial dilutions of purified recombinant Axl, MerTK or CD163 proteins were used to construct a standard curve. Blank wells were used to hold 1% BSA. For *in vitro* studies, cell culture supernatants were not diluted, and blank wells received serum-free X-VIVO 15 medium. Antigens were detected by a secondary biotin-conjugated antibody and horseradish peroxidase–conjugated streptavidin (BioLegend, San Diego, CA, USA). The plate was developed with 3,3′,5,5′-tetramethylbenzidine substrate. The reaction was stopped with 2 N sulfuric acid. Absorbance was detected at 450 nm and read with a reference wavelength set at 570 nm using a VersaMAX ELISA microplate reader (Molecular Devices, Sunnyvale, CA, USA). The optical density for each point was the average of duplicate samples. Concentrations were determined using SoftMax software (Molecular Devices) by applying four-parameter logistic regression to the standard curve. For sAxl quantitation, we used a mouse monoclonal anti-Axl Ab (clone 108724; R&D Systems) for capture, recombinant human Axl (R&D Systems) for the standard curve and a biotinylated goat polyclonal anti-Axl Ab (R&D Systems) for detection. For sMer quantitation, we used the Human Total Mer DuoSet IC (DYC891; R&D Systems) according to the manufacturer’s instructions. For sCD163 quantitation in plasma samples, we used the Human CD163 Quantikine ELISA Kit (DC1630; R&D Systems) according to the manufacturer’s instructions. For sCD163 quantitation in supernatants, we used a mouse monoclonal anti-CD163 Ab (clone EDHu-1; Novus Biologicals) for capture, recombinant human CD163 (R&D Systems) for the standard curve and a biotinylated goat polyclonal anti-CD163 Ab (R&D Systems) for detection.

### Flow cytometry

Membrane expression levels of Axl, MerTK and CD163 were measured in cultured monocytes after being washed in buffer containing 2% BSA. Monocytes were gated on the basis of forward and side light scatter and by using a phycoerythrin/cyanin 7 (PE/Cy7)-conjugated anti-CD14 antibody (BioLegend). The following mouse monoclonal antibodies were used for detection: PE-conjugated anti-MerTK (clone 125518; R&D Systems), PE-conjugated anti-Axl (clone 108724; R&D Systems) and allophycocyanin (APC)-conjugated anti-CD163 (clone GHI/61; BioLegend). Expression levels were evaluated using appropriate PE-labeled and APC-labeled isotype controls (BioLegend). Cells were analyzed using a FACSCalibur flow cytometer (BD Biosciences, San Jose, CA, USA) and FlowJo software (Tree Star, Ashland, OR, USA).

### Statistical analysis

Data are expressed as means ± SD. Comparisons of soluble receptor levels between patients and matched controls or between groups of patients with different laboratory or clinical characteristics were made using the Mann-Whitney *U* test. Correlations between soluble receptor levels and other continuous laboratory data were analyzed using Spearman’s rank correlation coefficient. Correlations of soluble receptor levels with the weighted scales of SLEDAI and the total BILAG index were made using Pearson’s correlation coefficient. Comparisons in soluble receptor levels among patients with inactive, moderately active or very active SLEDAI scores were made using one-way analysis of variance (ANOVA) with the Newman-Keuls multiple comparison test. For *in vitro* studies, differences between cell treatment groups were calculated using a paired Student’s *t*-test or one-way repeated-measures ANOVA (Newman-Keuls *post hoc* analysis) when more than two treatment groups were compared. Prism software (GraphPad Software, La Jolla, CA, USA) was employed for all analyses and graphing. A *P* value <0.05 was considered statistically significant.

## Results

### Plasma concentrations of sMer are increased in discrete SLE patient subsets compared to matched healthy controls

**Figure 1:**
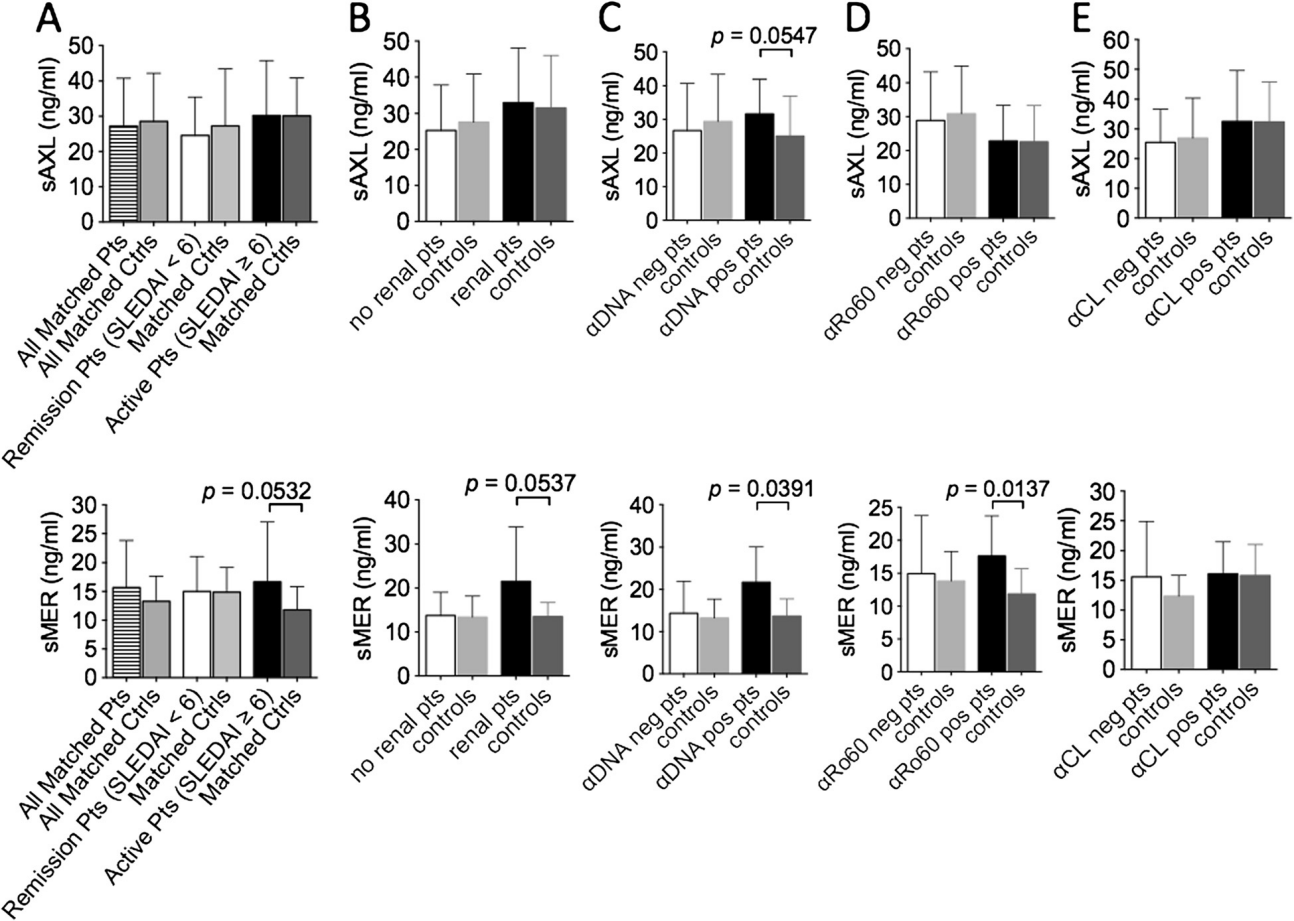
**Plasma levels of soluble Mer are increased in discrete subsets of systemic lupus erythematosus patients.** Levels of soluble Mer (sMer) or soluble Axl (sAxl) of 45 patients (pts) and 45 healthy controls (ctrls) were measured and compared by considering the whole groups **(A)** or by dividing patients and matched controls into subgroups according to disease activity (A), organ involvement **(B)** and autoantibody specificities **(C**, **D** and **E)**. αCL, anticardiolipin immunoglobulin G; αDNA, anti-double-stranded DNA; αRo60, anti-Sjögren’s syndrome antigen A/Ro 60 KDa; SLEDAI, Systemic Lupus Erythematosus Disease Activity Index.

We compared plasma levels of sAxl and sMer in 45 SLE patients and 45 age-, gender- and ethnicity-matched normal controls. No significant differences were observed between the two whole groups for either sAxl (27.21 ± 13.51 ng/ml vs. 28.56 ± 13.52 ng/ml) or sMer (15.68 ± 8.15 ng/ml vs. 13.30 ± 4.36 ng/ml) (Figure 1A). However, by subdividing patients with active disease (SLEDAI score ≥6) from those in remission (SLEDAI <6), we were able to note higher levels of sMer in the circulation of active patients compared to matched healthy individuals (16.67 ± 10.35 ng/ml vs 11.80 ± 4.04 ng/ml, respectively), although full statistical significance was not reached (*P* = 0.0532) (Figure 1A). Similarly, sMer concentrations were higher in patients with active renal disease (according to BILAG renal score) than in matched controls (21.53 ± 12.31 ng/ml vs. 13.51 ± 3.21 ng/ml; *P* = 0.0537) (Figure 1B). Significant increases in sMer levels compared to healthy controls were observed in patients with antibody positivity for anti-dsDNA (21.70 ± 8.36 ng/ml vs. 13.68 ± 4.08 ng/ml; *P* = 0.0391) (Figure 1C) and anti-Sjögren’s syndrome antigen A/Ro 60 kDa (SSA/Ro60) (17.64 ± 6.05 ng/ml vs. 11.86 ± 3.84 ng/ml; *P* = 0.0137) (Figure 1D). For sAxl, no differences were noted between the subsets of patients and matched controls, except for a trend toward higher levels in the circulation of anti-dsDNA-positive patients (31.64 ± 10.22 ng/ml vs. 25.05 ± 11.85 ng/ml; *P* = 0.0547) (Figure 1C). No differences were noted in plasma levels of sMer and sAxl compared to matched controls when considering patients with antiphospholipid antibodies (Figure 1E) or other anti-ENA autoantibodies or in patients with active hematological or vascular active disease (not shown).

### sAxl and sMer levels are associated with organ involvement, but only sMer correlates with SLE activity

**Figure 2:**
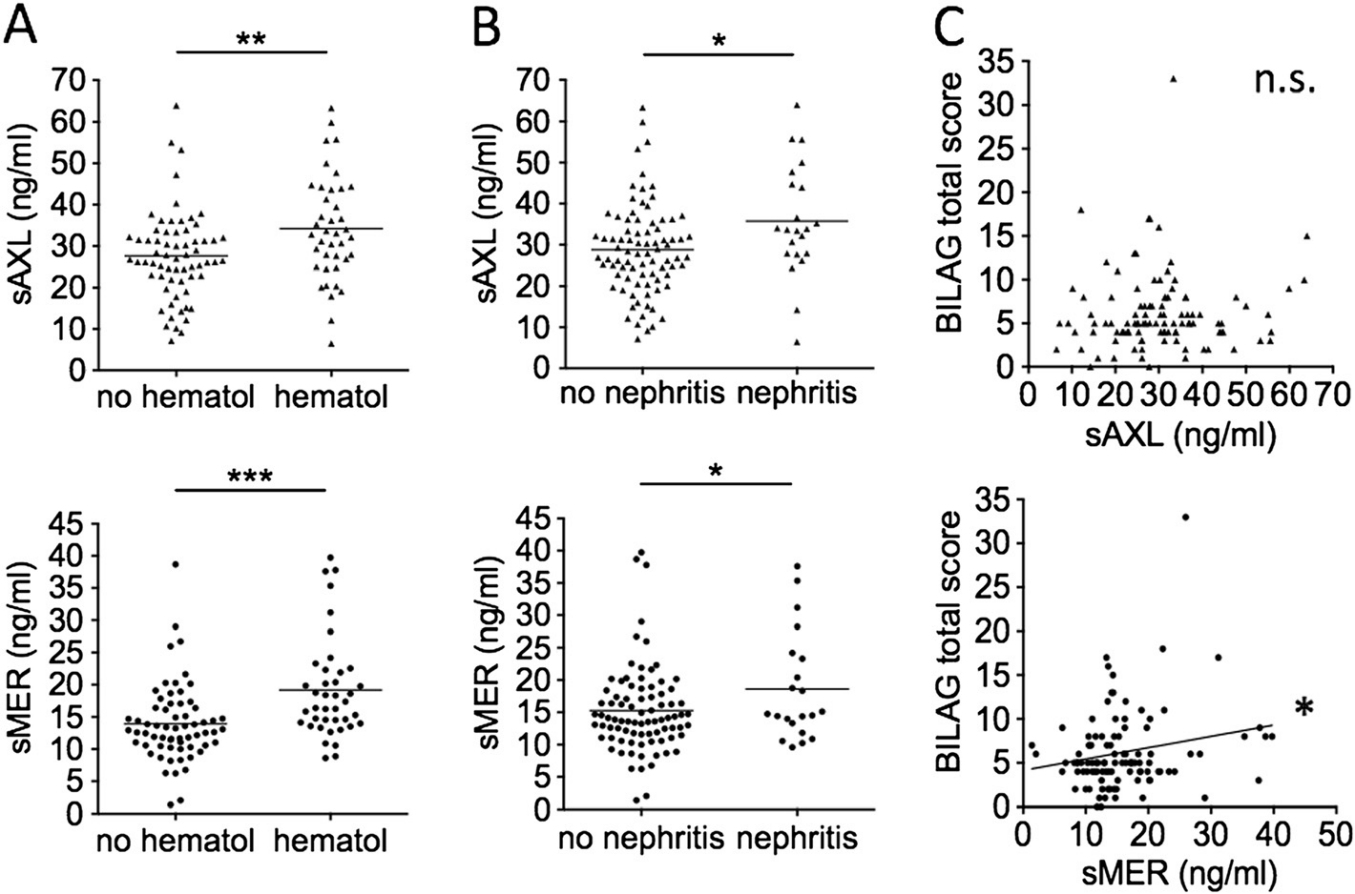
**Soluble Axl and soluble Mer levels are associated with British Isles Lupus Assessment Group index hematological and renal involvement, and soluble Mer is also associated with total BILAG score.** Levels of soluble Axl (sAxl) or soluble Mer (sMer) were measured in 107 matched and unmatched patients and compared according to active and/or stable (BILAG score ≥1) or inactive and/or absent (BILAG = 0) organ involvement **(A)** and **(B)**. Associations with total BILAG score were then calculated **(C)**. BILAG, British Isles Lupus Assessment Group index; hematol, hematological.*P < 0.05; **P < 0.01; ***P < 0.001; n.s., not significant.

We examined the entire cohort of 107 matched and unmatched SLE samples to look for potential differences in sAxl and sMer levels among patients according to organ involvement, laboratory parameters and clinical indices of lupus disease activity. We found that plasma concentrations of both sAxl and sMer were higher in patients with stable or active BILAG hematological involvement (that is, whose BILAG score was not zero) compared to patients with inactive or no hematological involvement (sAxl: 34.20 ± 12.72 vs 27.61 ± 10.62 ng/ml, *P* = 0.0057. sMer: 19.18 ± 7.85 vs. 13.92 ± 5.97 ng/ml, *P* = 0.0002) (Figure 2A), as well as in patients with stable or active BILAG renal involvement compared to patients with inactive or no renal involvement (sAxl: 35.75 ± 13.58 vs. 28.79 ± 10.99, *P* = 0.0115. sMer: 21.80 ± 11.33 vs. 15.42 ± 6.96 ng/ml, *P* = 0.0240) (Figure 2B). Concentrations of sMer levels, but not sAxl, directly correlated with BILAG total score (*r* = 0.20; *P* = 0.0387) (Figure 2C).

**Figure 3:**
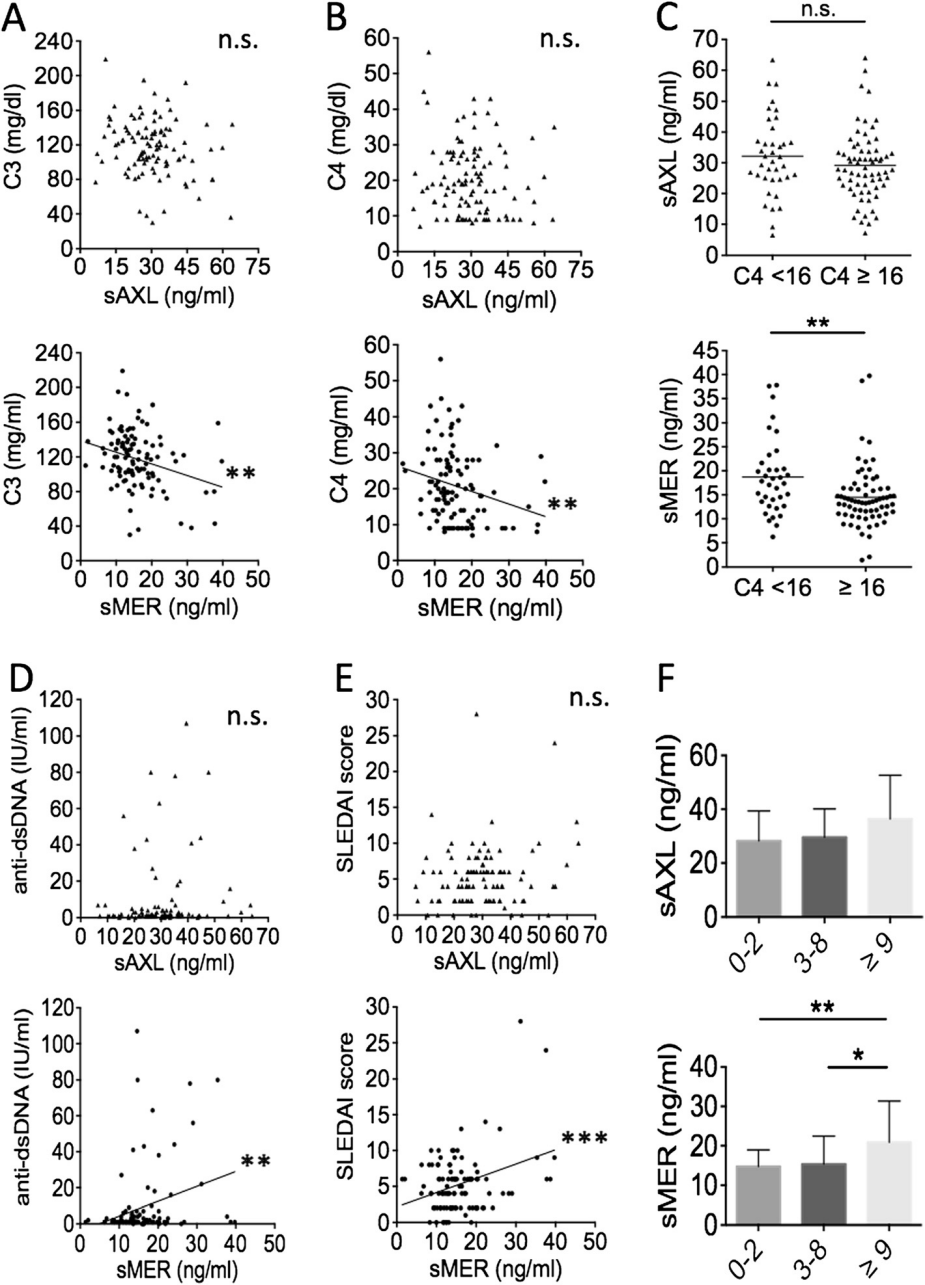
**Plasma levels of soluble Mer, but not soluble Axl, are closely associated with disease activity.** Levels of soluble Axl (sAxl) and soluble Mer (sMer) were analyzed according to levels of complement fraction 3 (C3) **(A)** and C4 **(B)** and **(C)**, circulating titers of anti-double-stranded DNA (anti-dsDNA) antibodies **(D)** and SLEDAI scores **(E)** and **(F)**. SLEDAI, Systemic Lupus Erythematosus Disease Activity Index. *P < 0.05; **P < 0.01; ***P < 0.001; n.s., not significant.

In fact, only for sMer did we find strong associations with lupus disease activity markers. Circulating levels of sMer were inversely correlated with C3 (*r* = −0.27; *P* = 0.0049) (Figure 3A) and C4 values (*r* = −0.25; *P* = 0.0053) (Figure 3B), whereas sAxl levels had only borderline relations with lower C3 levels (*r* = −0.17; *P* = 0.0840) (Figure 3A). In accord with these data, significantly higher levels of sMer were found in patients with a clear-cut reduction in C3 (22.24 ± 9.30 ng/ml vs. 14.79 ± 6.10 ng/ml; *P =* 0.0010 (not shown)) or in C4 (18.70 ± 7.91 ng/ml vs. 14.48 ± 6.32 ng/ml; *P =* 0.0029) (Figure 3C) according to our laboratory cutoff values (C3 <86 mg/dl and C4 <16 mg/dl). A less marked difference in sAxl values was noted between patients with low or normal C3 levels (36.76 ± 15.10 ng/ml vs. 28.99 ± 10.78 ng/ml; *P =* 0.0397 (not shown)), whereas no difference was observed between patients with low or normal C4 levels (32.19 ± 12.83 ng/ml vs. 29.14 ± 11.21 ng/ml) (Figure 3C). Moreover, levels of sMer, but not of sAxl, were directly correlated with titers of circulating anti-dsDNA autoantibodies (*r* = 0.29; *P* = 0.0032) (Figure 3D). Circulating levels of sMer showed a strong direct correlation with SLEDAI scores (*r* = 0.34; *P* = 0.0004) (Figure 3E), whereas only a trend could be detected for sAxl (*r* = 0.17; *P* = 0.0745). Accordingly, patients with very active disease (SLEDAI score ≥9) showed the highest levels of sMer in the circulation (20.88 ± 10.47 ng/ml), which was significantly different compared to patients with low or moderate disease activity (15.32 ± 7.08 ng/ml; *P <* 0.05) and even more significantly different from patients in complete remission (SLEDAI score <3, 14.74 ± 4.20 ng/ml; *P* < 0.01). No significant differences in sAxl levels were observed among these subsets of patients (Figure 3F).

### sAxl and sMer levels are associated with production of autoantibodies, but only sMer relates to lupus-specific autoimmunity

**Figure 4:**
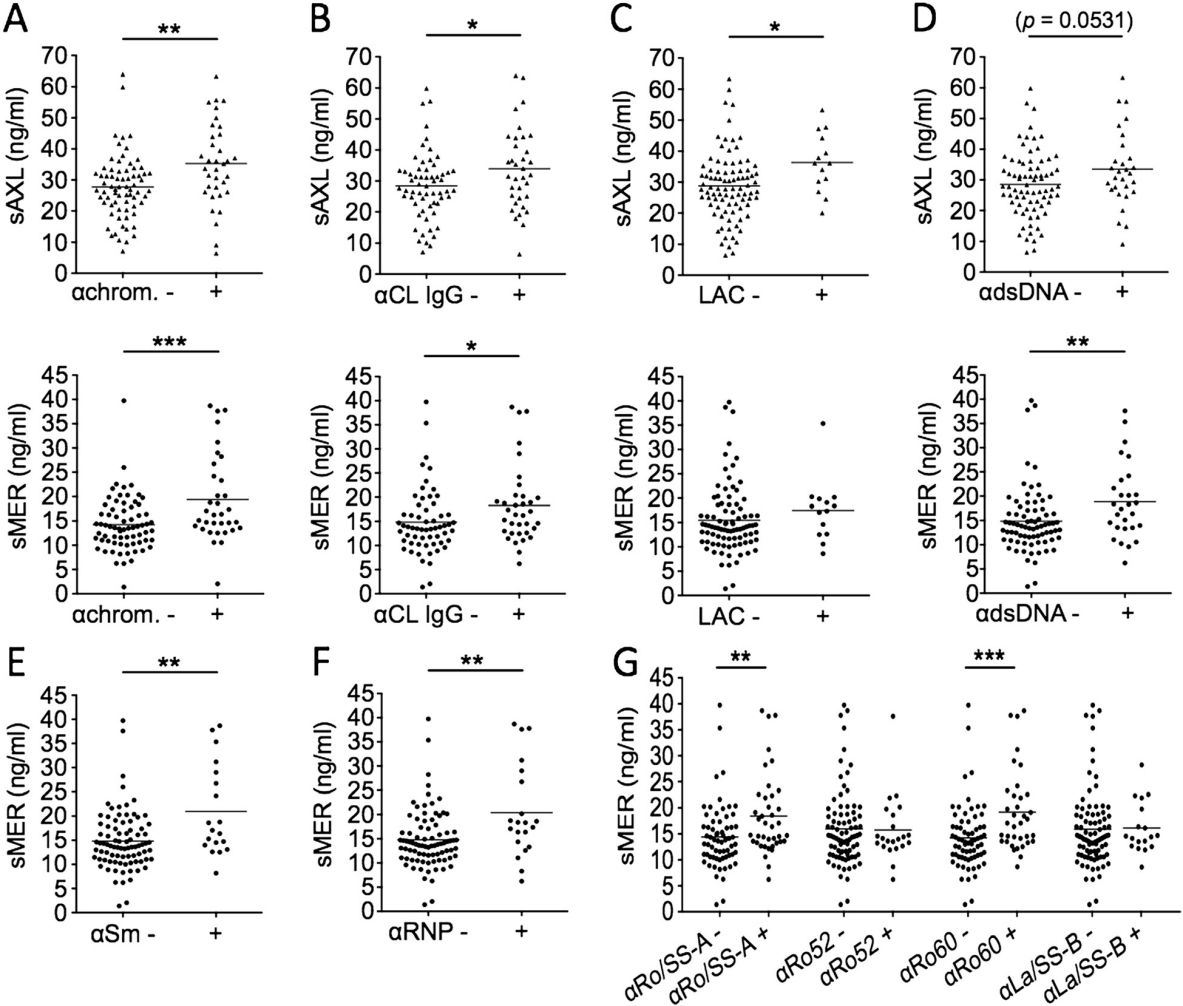
**Plasma levels of soluble Mer, but not soluble Axl, correlated with lupus autoantibody specificities.** Levels of soluble Axl (sAxl) and soluble Mer (sMer) were analyzed according to the presence or absence of the following antibodies: antichromatin (αchrom) **(A)**, anticardiolipin immunoglobulin G (IgG) (αCL) **(B)**, lupus anticoagulant (LAC) **(C)**, anti-double-stranded DNA (αdsDNA) **(D)**, anti-Smith (αSm) **(E)**, antiribonucleoprotein (αRNP) **(F)**, anti-Sjögren’s syndrome antigen A (SSA)/Ro 52 and 60 KDa (αRo52 and αRo60) and anti-Sjögren’s syndrome antigen B (SSB)/La **(G)**. *P < 0.05; **P < 0.01; ***P < 0.001.

Both sAxl and sMer were associated with the presence of circulating autoantibodies against nuclear material and phospholipids. Plasma concentrations of sAxl and sMer were higher in patients positive for antichromatin (sAxl: 35.28 ± 13.33 ng/ml vs. 27.74 ± 10.26 ng/ml; *P* = 0.0018. sMer: 19.41 ± 8.87 ng/ml vs. 14.26 ± 5.51 ng/ml; *P* = 0.0015) (Figure 4A) and anticardiolipin antibodies (sAxl: 33.92 ± 13.18 ng/ml vs. 28.42 ± 10.88 ng/ml; *P* = 0.0462. sMer: 18.27 ± 8.01 ng/ml vs. 14.83 ± 6.78 ng/ml; *P* = 0.0234) (Figure 4B). sAxl levels also correlated with lupus anticoagulant (LAC) positivity (36.36 ± 9.51 ng/ml vs. 29.19 ± 11.60 ng/ml; *P* = 0.0109) (Figure 4C). Nevertheless, only sMer levels were significantly higher in the presence of lupus-specific autoantibodies, such as anti-dsDNA (18.86 ± 7.78 ng/ml vs. 14.86 ± 6.65 ng/ml; *P* = 0.0092) (Figure 4D), anti-Smith (anti-Sm) (20.98 ± 9.40 ng/ml vs. 14.81 ± 6.08 ng/ml; *P* = 0.0064) (Figure 4E), antiribonucleoprotein (anti-RNP) (20.39 ± 9.52 ng/ml vs. 14.81 ± 6.01 ng/ml; *P* = 0.0070) (Figure 4F) and anti-Ro/SSA (18.42 ± 7.78 ng/ml vs. 14.40 ± 6.33 ng/ml; *P* = 0.0041) (Figure 4G). In particular, sMer levels were increased in patients positive for anti-Ro60 (19.19 ± 7.77 ng/ml vs. 14.23 ± 6.23 ng/ml; *P* = 0.0004), but not for anti-Ro 52 kDa autoantibodies (Figure 4G) and more specifically in those without concomitant positivity of anti-La/Sjögren’s syndrome antigen B (anti-La/SSB; 21.27 ± 9.08 ng/ml in patients with anti-Ro60 alone (*P* = 0.0006) vs. 16.58 ng/ml ± 4.85 ng/ml in patients positive for both anti-Ro60 and anti-La (*P* = 0.0575); not shown). A trend toward higher plasma levels of sAxl was observed in anti-dsDNA-positive patients (33.50 ± 12.47 ng/ml vs. 29.00 ± 11.44 ng/ml; *P* = 0.0535) (Figure 4D), whereas no difference could be detected in sAxl levels on the basis of anti-ENA autoantibody positivity (not shown).

### Concentrations of sAxl and sMer are differentially correlated with levels of Gas6 and ProS

**Figure 5:**
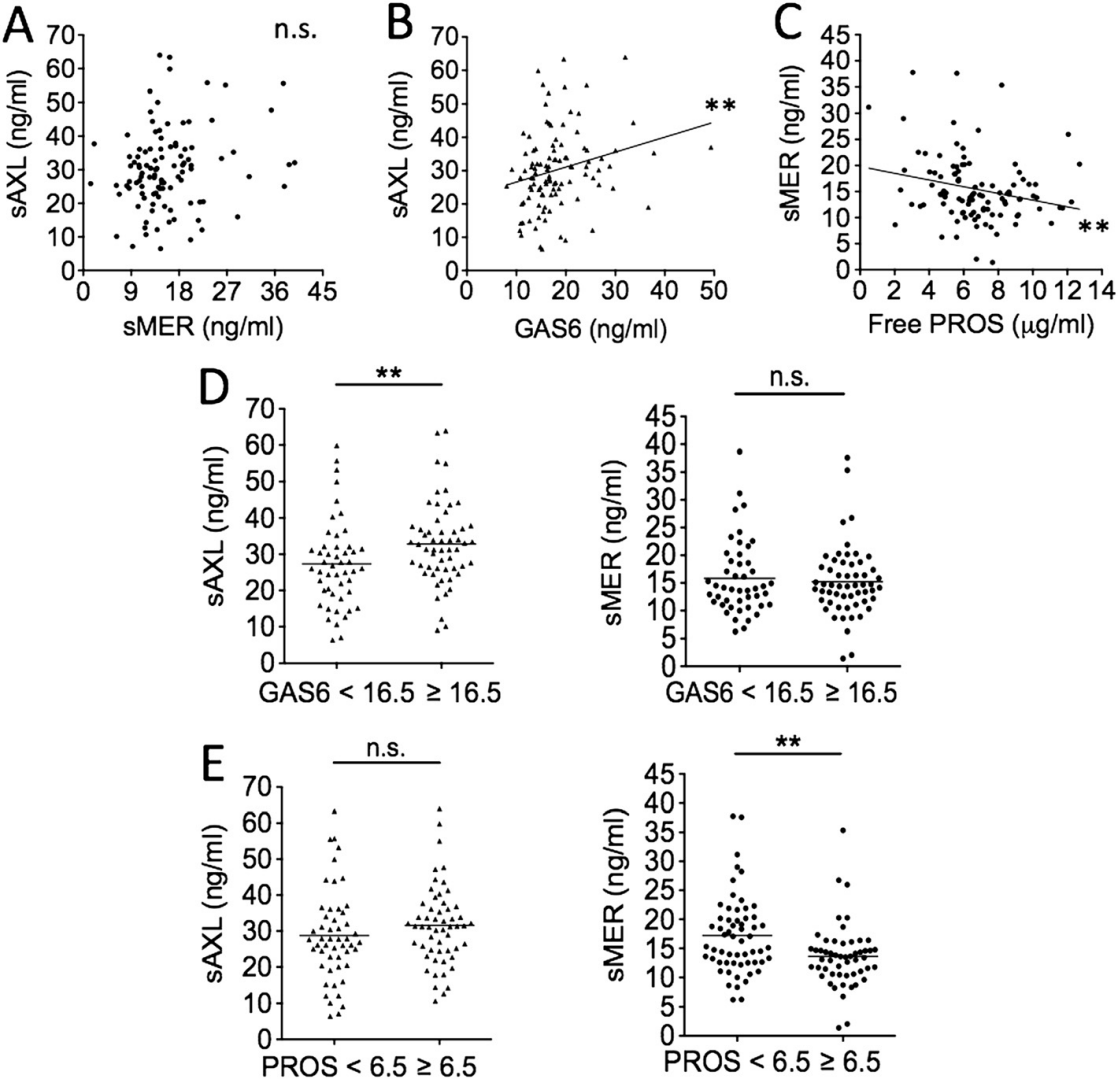
**Plasma levels of soluble Axl correlated with growth arrest–specific 6, but soluble Mer correlated with reduced free Protein S.** Levels of soluble Axl (sAxl) and soluble Mer (sMer) were related to each other **(A)** and to Tyro3, Axl and MerTK (TAM) receptor ligands growth arrest–specific 6 (Gas6) and free Protein S (ProS) **(B**, **C**, **D**, and **E)**. Cutoff values of Gas6 (ng/ml) and ProS (μg/ml) were established by considering mean values in patients and controls (Table 1). **P < 0.01; n.s., not significant.

Correlation between sAxl and sMer plasma levels did not reach full statistical significance in SLE patients (*r* = 0.18; *P* = 0.0675) (Figure 5A). We found significant correlations between concentrations of sAxl and sMer, as well as plasma levels of their ligands Gas6 and free ProS, previously measured in the same cohort [24]. However, sAxl and sMer correlated differently with Gas6 and ProS. sAxl was directly correlated with Gas6 levels (*r* = +0.31; *P* = 0.0011) (Figure 5B), but sMer was inversely correlated with free ProS levels (*r* = −0.26; *P* = 0.0088). Accordingly, a significant difference in concentrations of Axl, but not of sMer, was found between patients with high vs. low Gas6 levels (32.86 ± 11.33 ng/ml vs. 27.33 ± 11.95 ng/ml; *P* = 0.0050) (Figure 5D), whereas a significant difference in the concentrations of sMer, but not of sAxl, was found between patients with high or low free ProS (13.64 ± 5.43 ng/ml vs. 17.21 ± 6.68 ng/ml; *P* = 0.0021) (Figure 5E). Cutoff values of Gas6 and free ProS (16.5 ng/ml and 6.5 μg/ml, respectively) were established according to their mean values among patients and matched healthy controls (Table 1). In healthy controls, we failed to find significant correlations (not shown).

### sMer is an M2c activation marker, whereas sAxl is a type-I IFN stimulation marker

We investigated whether the release of sMer and sAxl was related to discrete immunological phenotypes of monocytes/macrophages. For this purpose, we measured concentrations of sMer and sAxl in supernatants of monocytes/macrophages cultured in the presence of medium alone (M0), IFN-γ or GM-CSF (M1), IL-17 (M17), IL-4 (M2a), IL-10, M-CSF, M-CSF plus IL-10, or glucocorticoids (M2c), transforming growth factor β (TGF-β) and combinations of M2 cytokines such as TGF-β plus IL-4 or IL-4 plus IL-10 (other M2 activation states).

**Figure 6:**
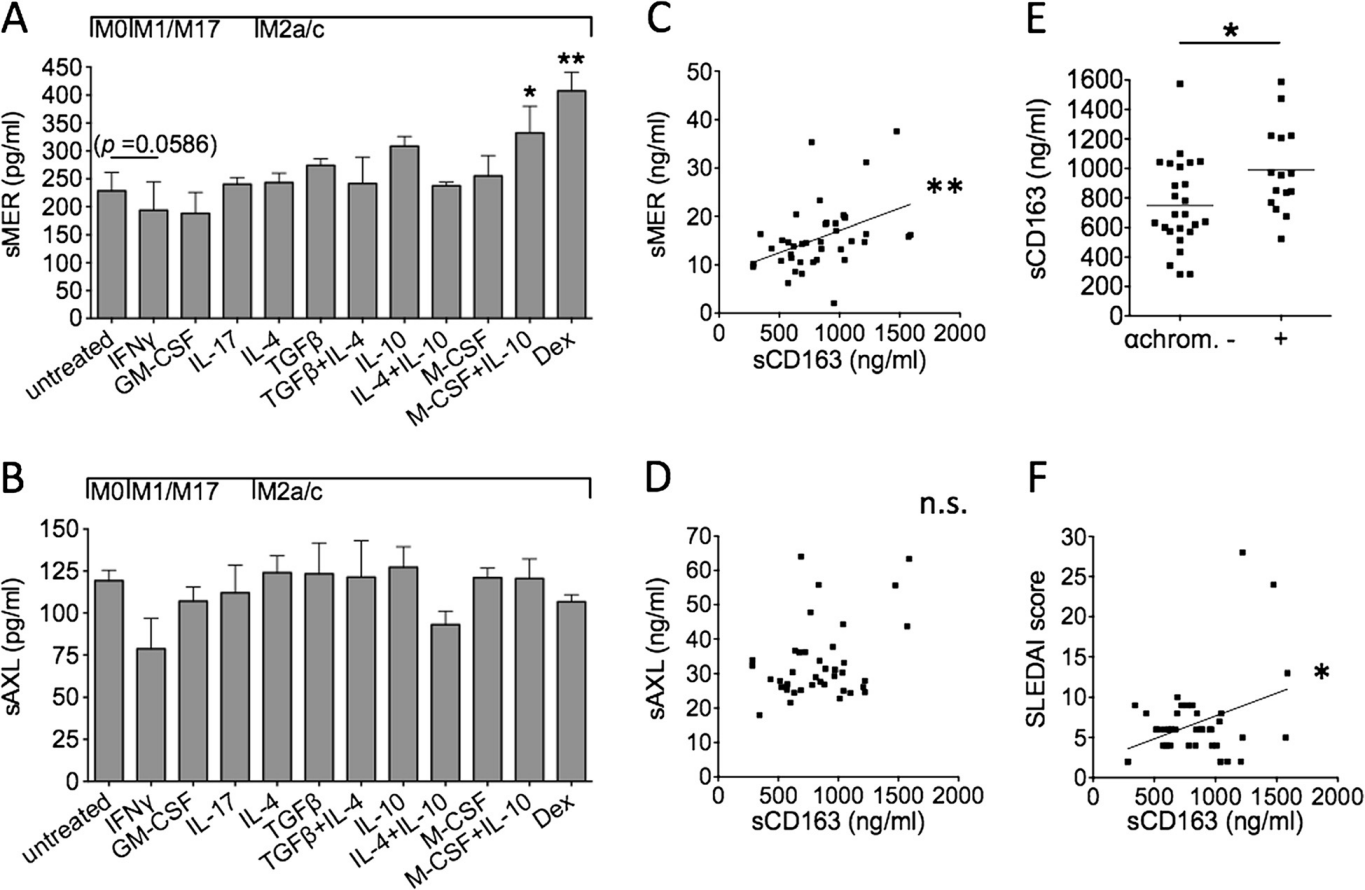
**Soluble Mer is released by M2c monocytes/macrophages and correlates with circulating levels of soluble CD163 in systemic lupus erythematosus, and soluble CD163, like soluble Mer, correlates with Systemic Lupus Erythematosus Disease Activity Index score.** Levels of soluble Mer (sMer) **(A)** and soluble Axl (sAxl) **(B)** were measured in supernatants of monocytes/macrophages cultured for 3 days in the presence of medium alone (resting M0), interferon γ (IFN-γ), granulocyte macrophage colony-stimulating factor (GM-CSF), interleukin 17 (IL-17) (classically activated and/or proinflammatory M1 and M17), IL-4, transforming growth factor β (TGF-β), IL-10, macrophage colony-stimulating factor (M-CSF), dexamethasone (Dex), alone or in combination (alternatively activated and/or regulatory M2a and M2c cells). In 40 consecutive patients in our cohort, potential correlations between plasma levels of sMer **(C)** or sAxl **(D)** and soluble CD163 (sCD163) were examined. Levels of sCD163 were also analyzed according to circulating autoantibodies **(E)** and disease activity **(F)**. αchrom., antichromatin; SLEDAI, Systemic Lupus Erythematosus Disease Activity Index. *P < 0.05; **P < 0.01; n.s., not significant.

We found that sMer was abundantly released by M2c cells, driven by M-CSF plus IL-10 or by glucocorticoids (dexamethasone). A slight decrease in sMer levels was noted in the supernatants of M1 cells, driven by IFN-γ (Figure 6A). By contrast, concentrations of sAxl were not significantly influenced by either M1 or M2 polarization (Figure 6B).

In 40 consecutive SLE patients, we looked for potential relations between either sMer or sAxl levels and plasma concentrations of sCD163, a well-known marker of M2c cell activation. In accord with the *in vitro* data, we found that circulating levels of sMer were strongly associated with plasma concentrations of sCD163 (*r* = 0.50; *P* = 0.0011) (Figure 6C), whereas no significant correlation was observed between sAxl and sCD163 levels (r = +0.17; *P* = 0.2978) (Figure 6D). Levels of sCD163 were associated with positivity of antichromatin autoantibodies (988.22 ng/ml ± 299.05 ng/ml vs. 747.34 ng/ml ± 300.38 ng/ml; *P* = 0.0255) (Figure 6E), and, as observed for sMer, correlated with lupus disease activity as assessed by SLEDAI score (*r* = 0.35; *P* = 0.0257) (Figure 6F).

Because SLE is characterized by the so-called “interferon signature” [25,26], we subsequently examined the role of type I IFNs in regulating the release of sAxl, sMer and sCD163 and looked at the effects of combining type I IFNs (IFN-α and IFN-β) with macrophage growth factors (M-CSF and GM-CSF) and/or with M2c polarizing agents (IL-10 or dexamethasone). Ectodomain shedding of membrane receptors and consequent release of soluble receptors was triggered by using low doses of LPS.

**Figure 7:**
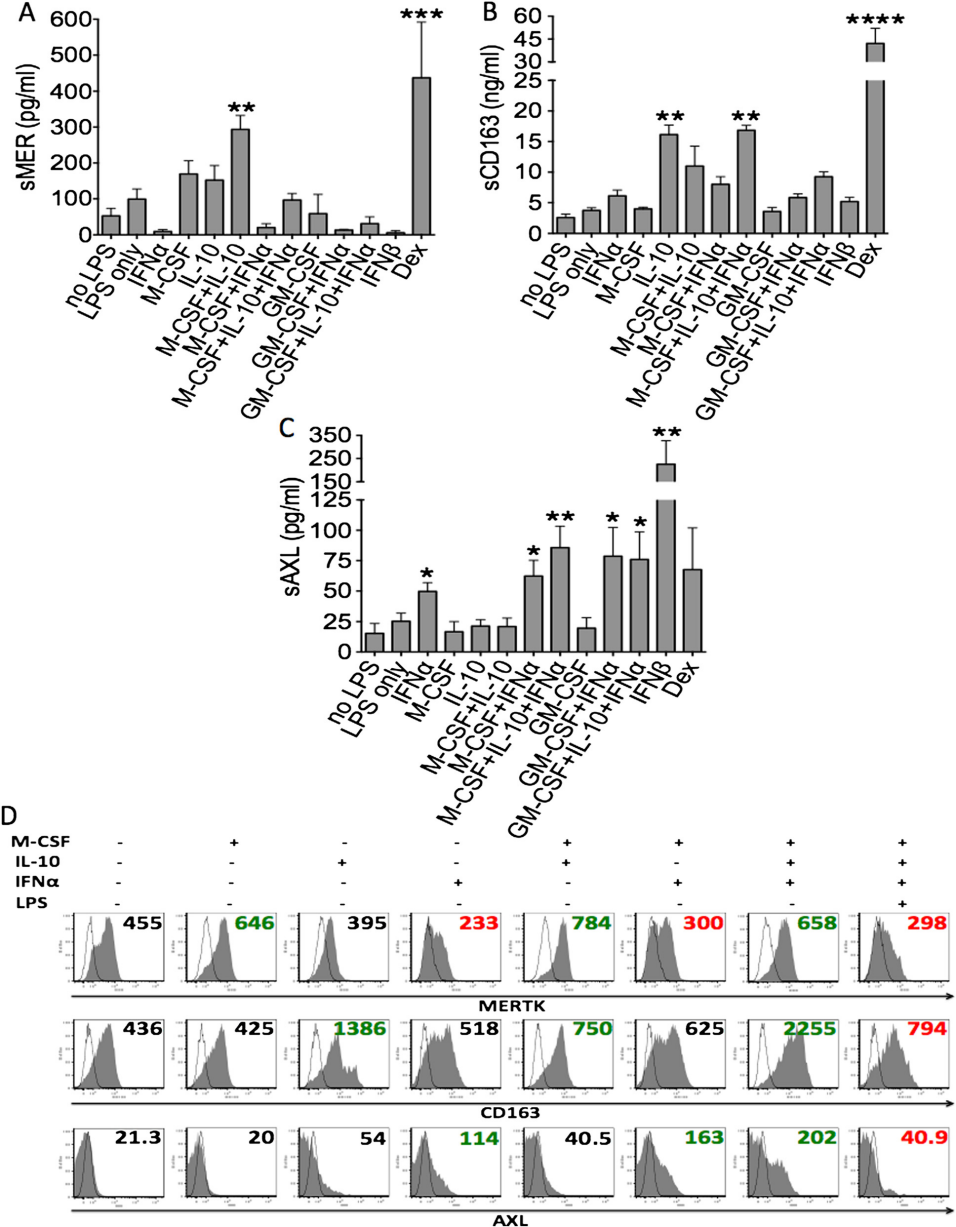
**Interferon α/β is required for production of soluble Axl, and combining M2c polarizing conditions with type I interferon exposure inhibits soluble Mer production and enhances soluble Axl release.** Levels of soluble Mer (sMer) **(A)**, soluble CD163 (sCD163) **(B)** and soluble Axl (sAxl) **(C)** were measured in supernatants of monocytes/macrophages cultured for 3 days in the presence or absence of type I interferons (IFN-α or IFN-β), macrophage growth factors (macrophage colony-stimulating factor (M-CSF) or granulocyte macrophage colony-stimulating factor (GM-CSF)), M2c polarizing agents (interleukin 10 (IL-10) or dexamethasone) or combinations of these. On day 2, low doses of lipopolysaccharide (LPS) were added for an additional 24 hours to stimulate ectodomain shedding of membrane receptors. Surface expression levels of Mer receptor tyrosine kinase (MerTK), Axl and CD163 were measured by flow cytometry **(D)**. Numbers shown in (D) refer to mean fluorescence intensity values. Data are representative of three independent experiments. Dex, dexamethasone. *P < 0.05; **P < 0.01; ***P < 0.001; ****P < 0.0001.

Release appeared to be moderately enhanced in the presence of LPS alone, although no statistically significant difference was reached (Figure 7A to C). Levels of sMer and sCD163 were confirmed to be highest upon exposure to M2c polarizing stimuli. M-CSF was required in combination with IL-10 to enhance sMer production, but it was not required for sCD163. Stimulation with either IFN-α or IFN-β alone failed to exert significant effects on either sMer or sCD163 levels (Figures 7A and B).

By contrast, both IFN-α and IFN-β were found to stimulate significant production of sAxl, with IFN-β stimulating the highest levels. The addition of M-CSF or GM-CSF enhanced IFN-α effects, although the differences did not reach full statistical significance. Stimulation with M2c agents alone did not influence sAxl production (Figure 7C).

Combining M2c polarizing conditions (M-CSF plus IL-10) with IFN-α exposure had variable effects. IFN-α reduced sMer production induced by M-CSF plus IL-10 (Figure 7A). Conversely, M-CSF plus IL-10 enhanced sAxl production induced by IFN-α (Figure 7B). IFN-α neutralized the modulatory effect of M-CSF on sCD163 production induced by IL-10 (Figure 7C).

Release of soluble ectodomains mirrored membrane expression patterns of the respective receptors MerTK, Axl and CD163 in the absence of LPS. M-CSF, IL-10 and dexamethasone induced MerTK and CD163 expression, and IFN-α stimulated Axl expression. Furthermore, IFN-α reduced MerTK upregulation driven by M-CSF plus IL-10, whereas M-CSF plus IL-10 enhanced Axl upregulation driven by IFN-α. IFN-α neutralized the modulatory effect of M-CSF on CD163 upregulation driven by IL-10. Consistent with its role as a sheddase agonist [17,19,20,27], LPS decreased surface expression of MerTK and CD163 induced by M-CSF, IL-10 and dexamethasone, as well as surface expression of Axl induced by IFN-α (Figure 7D).

## Discussion

SLE is characterized by impaired macrophage phagocytosis of ACs [1], delayed and proinflammatory AC clearance [2,3] and increased cellular expression of the type I IFN-inducible gene spectrum: the so-called IFN “signature” [25,26]. All these events reflect and contribute to aberrant stimulation of innate immunity. The family of the TAMRs acts to impede such events, thereby preventing systemic autoimmunity. In particular, MerTK is key to efficient clearance of early ACs and to macrophage production of anti-inflammatory cytokines [9-14], and Axl is primarily involved in feedback pathways controlling type I IFN-mediated innate immune activation [15-17]. In the present study, we analyzed the levels of sAxl and sMer receptors in the circulation of SLE patients and investigated potential relations with the clinical, laboratory and immunological aspects of the disease.

We found that increased levels of both sMer and sAxl are associated with general traits of systemic immunity, such as antinuclear and antiphospholipid autoantibody positivity. Additionally, both correlated with hematologic and renal involvement. Nevertheless, we found that sMer, but not sAxl, was significantly associated with lupus-specific humoral autoimmune responses, which were characterized by production of anti-dsDNA, anti-Sm, anti-RNP and anti-Ro60 autoantibodies. Remarkably, only sMer showed strong correlations with disease activity indices, such as C3 and C4 reduction, circulating titers of anti-dsDNA and SLEDAI and total BILAG scores. Compared to matched healthy controls, plasma levels of sMer, but not sAxl, were found to be higher in patients with active lupus (SLEDAI score ≥6), active BILAG renal score and anti-dsDNA and anti-Ro60 positivity. The highest values of sMer were observed in patients with very active lupus (SLEDAI score ≥9). Differences between sAxl and sMer also included relations with their ligands, Gas6 and ProS. In particular, sAxl directly correlated with Gas6 levels, whereas sMer correlated with reduced levels of free ProS. Notably, we found that sAxl and sMer were produced by different immune phenotypes of monocytes/macrophages. sAxl release was induced in the presence of either IFN-α or IFN-β, and sMer was released by M2c differentiated cells, similarly to what we observed for sCD163, a well-known marker of M2 activation. In fact, concentrations of sMer in the circulation of lupus patients directly correlated with plasma levels of sCD163, and sCD163, similarly to sMer, significantly correlated with disease activity. Combining type I IFN exposure with M2c polarizing conditions reduced M2c-driven sMer production while increasing IFN-α-induced sAxl release. The prototypical T-helper cytokines IFN-γ, IL-4 and IL-17 did not exert significant influences on either sAxl or sMer production.

To the best of our knowledge, herein we describe for the first time sMer as a biomarker of M2c activation, in parallel with sCD163. We confirmed the correlation between SLEDAI scores and plasma levels of sMer reported by Wu *et al.*[28] and Recarte-Pelz *et al.*[29]. We also have shown a direct correlation of sMer with sCD163 levels and a significant correlation between SLEDAI and sCD163 levels. Our data strongly suggest a strict relation between SLE activity and M2c homeostasis, in agreement with recent data from Nakayama *et al.* showing sCD163 associations with anti-dsDNA positivity and leukopenia [30]. Similarities between sMer and sCD163, with regard to their expression patterns and their associations in SLE, are consistent with the fact that their respective membrane receptors MerTK and CD163 are both upregulated on the surface of regulatory M2c monocytes/macrophages [9]. Both are cleaved by the same metalloproteinase, ADAM-17 [20,27], in contrast to sAxl, which is cleaved by ADAM-10 [18]. Both MerTK and CD163 serve to trigger IL-10 release from M2c cells [9,31], and both protect macrophages from oxidative stress and subsequent apoptosis induced by hydrogen peroxide, oxidized lipoproteins or iron-containing heme [32-34].

The biological significance of sMer and sCD163 in SLE can be construed as due to at least two mechanisms. Correlations of sMer and sCD163 with SLE activity may indicate a compensatory increase in M2c activation and turnover of monocytes and/or macrophages, with the aim of promoting efferocytosis and immune regulation in response to the still poorly defined inflammatory triggers and to the increased rates of apoptosis. Alternatively, excess ectodomain shedding of MerTK and CD163 by ADAM-17 may account for a functional impairment of M2c monocytes/macrophages and could itself contribute to chronic inflammation, defective clearance of early ACs and autoimmunity. It is known, in fact, that TAMR-knockout mice develop hyperreactive immune responses and severe lymphoproliferation [11,35]. In particular, disrupted MerTK expression is associated with a SLE-like syndrome in mice [36], and gene polymorphisms of MerTK and Gas6 are associated with clinical manifestations in SLE patients [37,38]. Besides gene defects and polymorphisms, posttranslation inhibition of these molecular pathways through ectodomain shedding may affect efferocytosis and regulatory responses [19], thus favoring accumulation of AC-derived autoantigens. ADAM metalloproteinases are, in fact, activated upon multiple conditions, including infections, oxidative stress and paracrine signals [18-20,27,39-42]. Of note, ADAM-17 is also known to cleave and inhibit the membrane receptor for M-CSF [43], which is needed for complete M2c differentiation [9]. Besides its crucial role in promoting macrophage release of major proinflammatory mediators, including TNF-α and IL-6 [40], ADAM-17 may thus exert its proinflammatory effects by interfering with differentiation and activity of regulatory M2c macrophages. From this perspective, impeding ectodomain shedding by the use of safe and selective ADAM inhibitors might help to restore macrophage homeostasis in SLE [44].

Cleavage of Axl into sAxl may in turn alter the homeostatic mechanisms regulating TLR-mediated activation [15,16], thus resulting in exaggerated production of IFN-α in response to AC-derived autoantigens. Excess activation of TLR/IFN pathways may ultimately lead to dendritic cell maturation, presentation of autoantigens to autoreactive T cells, chronic B-cell activation, oligoclonal expansion of plasmablasts and production of autoantibodies [26]. In addition, both sMer and sAxl are able to sequester the ligand Gas6 [18,19,45], thus interfering with membrane TAMR-induced regulatory signaling. Contrary to Ekman *et al.*[46], however, we could not confirm a significant association between SLEDAI scores and plasma levels of sAxl. Similarly, Recarte-Pelz *et al.*[29] failed to find such an association. The discrepancy might be due to differences between patient populations or to the use of different detection reagents. The same ELISA kit (R&D Systems) was used by Wu *et al.*[28], Recarte-Pelz *et al.*[29] and our laboratory for detection of sMer in SLE patients. For sAxl, instead, Ekman *et al.*[46] used an ELISA type developed in their laboratory, whereas we and Recarte-Pelz e*t al.*[29] used the same commercially available anti-Axl detection antibody (R&D Systems). The weaker association with SLE activity of sAxl compared to sMer suggests a more indirect role of sAxl in SLE pathogenesis. Whereas the cleavage of MerTK may be critical for the accumulation of AC-derived autoantigens and production of pathogenic lupus-specific autoantibodies, the cleavage of Axl could be more generally related to uninhibited TLR activation and production of IFN-α/β and other proinflammatory cytokines. Consistent with this view, sAxl, but not sMer, was found to be increased in nonautoimmune inflammatory diseases such as critical limb ischemia [28,47].

Among lupus-specific autoantibodies, sMer levels showed the strongest association with anti-Ro60 antibodies, particularly in the absence of a concomitant anti-La positivity. No association was found with anti-Ro52 antibodies. In fact, serum positivity for anti-Ro60 best discriminated patients with significantly higher levels of sMer compared to matched healthy controls. It is noteworthy that Ro60 is translocated to the cell surface of ACs during early apoptosis independently of La and Ro52 [48]. Autoantibodies against surface-exposed Ro epitopes are specific for a subset of SLE patients showing positivity of anti-Ro60 without anti-La, whereas double-positivity of anti-Ro60 and anti-La is consequent to intermolecular spreading from Ro to La, in which antigens are exposed on late ACs or released from necrotic ACs [48]. Anti-Ro52 antibodies are instead more prevalent in conditions other than SLE, such as primary Sjögren’s syndrome and idiopathic inflammatory myopathies [49]. Because production of anti-Ro60 antibodies represents a lupus-specific humoral autoimmune response against early ACs [48] and MerTK is specifically required for M2c macrophage phagocytosis of early ACs [9], the strong association that we found between anti-Ro60 and sMer in SLE patients might reflect a compensatory increase in M2c activation of monocytes and/or macrophages to enhance the clearance of early ACs by MerTK. Alternatively, the accumulation of early ACs fostering anti-Ro60 production might be itself a consequence of excess ectodomain shedding of MerTK, which would interfere with the clearance efficiency of M2c cells. The latter hypothesis suggests a putative role for the cleavage of MerTK in SLE pathogenesis, at least in a subgroup of anti-Ro60-positive patients.

Our data pertaining to the relation of sAxl and sMer to Gas6 and ProS levels are consistent with previous data on receptor-ligand binding affinity. Correlation between sAxl and Gas6 is in fact consistent with the tenfold higher binding affinity of Gas6 to Axl than to MerTK [50], as well as with the previous finding that Gas6 is primarily complexed with sAxl in human blood [45]. Correlation between sMer and reduced free ProS levels is consistent with the fact that ProS binds to MerTK [50], whereas no connection between ProS and Axl has been demonstrated to date. ProS serves as the main bridging molecule between phosphatidylserine on ACs and MerTK on the surface of human monocytes and/or macrophages [8]; however, whether sMer binds to ProS remains to be established. ProS needs to oligomerize to bind to Mer by interacting with other molecules of ProS on phosphatidylserine-containing surfaces [51]. Intriguingly, ProS is also able to bind to microparticles, besides ACs [52]. Plasma microparticles and/or circulating ACs may therefore serve as a scaffold for ProS oligomerization in circulation. In SLE, levels of microparticles increase with disease activity [53], whereas levels of free ProS decrease with disease activity [24]. It is tempting to speculate that, in active SLE patients, ProS may bind to microparticles, thus provoking reduction in free ProS levels, ProS oligomerization and potential formation of ProS-sMer complexes. In support of this view, it has been shown that HIV-infected patients also show reduced free ProS levels, and this reduction has been related to ProS binding to circulating microparticles [54]. Further investigation is needed to address this hypothesis.

## Conclusions

sMer and sCD163 are valuable biomarkers of M2c activation and disease activity in SLE. Increased levels of sMer in SLE are also associated with lower levels of free ProS and lupus-specific humoral immune response. sAxl is associated with type I IFN stimulation, correlates with Gas6 levels and shows minor associations with SLE activity and autoimmunity. Our data suggest a link between homeostasis of efferocytic and anti-inflammatory M2c (CD163^+^ and MerTK^+^) monocytes/macrophages and the pathogenesis of human SLE.

## Abbreviations

AC: Apoptotic cell; ADAM: A disintegrase and metalloproteinase; APC: Allophycocyanin; BILAG: British Isles Lupus Assessment Group Index; BSA: Bovine serum albumin; CD: Cluster of differentiation; Dex: Dexamethasone; ELISA: Enzyme-linked immunosorbent assay; GM-CSF: Granulocyte macrophage colony-stimulating factor; HRP: Horseradish peroxidase; IFN: Interferon; IL: Interleukin; LPS: Lipopolysaccharide; M-CSF: Macrophage colony-stimulating factor; PBS: Phosphate-buffered saline; PE: Phycoerythrin; PE/Cy7: Phycoerythrin with cyanin 7; PMA: Phorbol 12-myristate 13-acetate; ProS: Protein S; sAXL: Soluble AXL; sCD163: Soluble CD163; SLE: Systemic lupus erythematosus; SLEDAI: Systemic Lupus Erythematosus Disease Activity Index; sMER: Soluble Mer; TAMR: Tyro3/AXL/Mer receptor family; TLR: Toll-like receptor; TNF-α: Tumor necrosis factor α.

## Competing interests

The authors declare that they have no competing interests.

## Authors’ contributions

GZ contributed to the conception and design of the study; performed experiments; collected, analyzed and interpreted data; and wrote the manuscript. JG and LMD performed experiments and collected data. JTM provided plasma samples and clinical characterization of the Oklahoma Cohort for Rheumatic Diseases. PLC conceived, designed and coordinated the study; interpreted data; and critically revised the manuscript. All authors approved the final version of the manuscript.
